# Assessment of Truflex™ articulating stylet versus conventional rigid Portex™ stylet as an intubation guide with the D-blade of C-Mac™ videolaryngoscope during elective tracheal intubation: study protocol for a randomized controlled trial

**DOI:** 10.1186/1745-6215-14-298

**Published:** 2013-09-16

**Authors:** Aida Al-Qasmi, Wafa Al-Alawi, Azharuddin Mohammed Malik, Rashid Manzoor Khan, Naresh Kaul

**Affiliations:** 1 Department of Anesthesia & ICU, Khoula Hospital, Muscat, Sultanate of Oman; 2 Department of Medicine, J N Medical College, Aligarh, India

**Keywords:** Videolaryngoscope, Tracheal intubation, Truflex stylet

## Abstract

**Background:**

A variety of videolaryngoscopes with angulated blade have been recently introduced into clinical practice. They provide an indirect view of the glottic structures in normal and challenging clinical settings. Despite the very good visualization of the laryngeal structures by these devices, the insertion and advancement of the endotracheal tube may be prolonged and occasionally fail as it does not conform to the enhanced angulation of the blade. To overcome this handicap, it is recommended to use a pre-shaped, styleted tracheal tube during intubation. Unfortunately, these malleable rigid stylets permit only a fixed shape to the advancing endotracheal tube. This may necessitate withdrawal of endotracheal tube-stylet assembly for reshaping, before undertaking a new attempt. This may cause soft tissue injury and hemodynamic disturbance.

This single-blinded randomized clinical trial aims to overcome these handicaps using a novel method of dynamically changing the shape of the advancing endotracheal tube by Truflex™ articulating stylet as per need during D-blade C-Mac™ videolaryngoscopy.

**Methods:**

One hundred and fifty four patients between 18 and 60 years of age belonging to either sex undergoing tracheal intubation under uniform general anesthetic technique will be randomly divided into Portex™ malleable stylet group and Truflex™ articulating stylet group. The primary efficacy variable of success/failure between the two groups will be analyzed using the chi square test. For comparison of intubation times and the Intubation Difficulty Score, ANOVA will be used. Primary efficacy endpoint results will be successful or failed tracheal intubation in the first attempt, total intubation time and the intubation difficulty score. Secondary efficacy endpoints will be overall user satisfaction graded from 1 to 10 (1 = very poor, 10 = excellent), Cormack and Lehane’s grading, glotticoscopy time and ETT negotiation time and total number of intubation attempts. Result of safety endpoints will include dental and airway trauma, hemodynamic disturbances, arrhythmias or cardiac arrest.

**Trial registration:**

Current Controlled Trials ISRCTN57679531; Date of registration 12/02/2013

## Background

**Figure 1:**
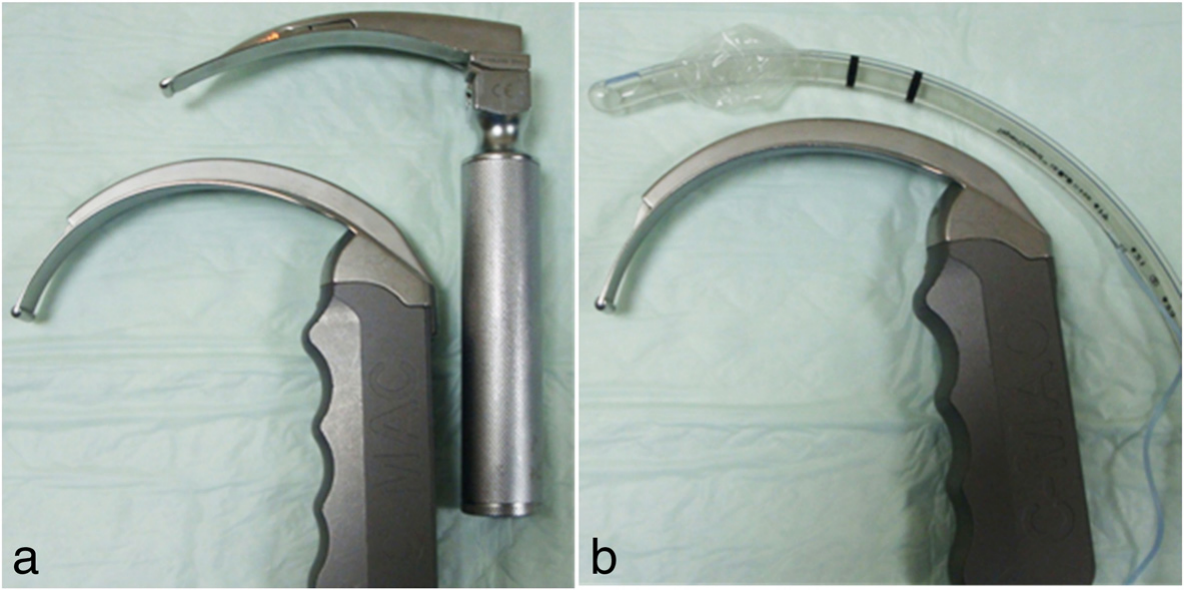
**Enhanced angulation of the D-blade of the C-Mac™ videolaryngoscope. (a)** Shown is the sharper blade angulation of the D-blade of the C-Mac™ videolaryngoscope versus the conventional Macintosh laryngoscope blade. **(b)** The natural curvature of the traditional endotracheal tube does not match the enhanced angulation of the D-blade of the C-Mac™ videolaryngoscope.

Videolaryngoscopes now play an important role as an alternative to conventional rigid laryngoscopy. A major advantage of the videolaryngoscope is better visualization of the larynx [1,2]. This is achieved as a result of increased angulation of the blade compared to the standard Macintosh blade (Figure 1a). Videolaryngoscopes provide an indirect view of the laryngeal structures on the screen compared to the traditional Macintosh laryngoscope, which necessitates in-line visualization of the glottis using the naked eye for successful tracheal intubation. Videolaryngoscopes serve as a valuable teaching aid as they display the laryngeal structure on a video screen [3-6]. Both reusable and disposable versions are now available.

Despite several advantages offered by the videolaryngoscopes, their major handicap is their enhanced anterior angulation of the blades, such as that of the Glide-Scope™ (Verathon Medical, Bothell WA, USA), the McGrath series 5 (Aircraft Medical, Edinburgh, UK), and the TruView™ PCD devices (Truphatek International Limited, Netanya, Israel), which makes viewing of the laryngeal structure easier [1,2], but negotiation of the endotracheal tube (ETT) towards the glottis difficult, and at times a failure [7,8]. This is attributed to the fact that the tip of the ETT has to pass around the steep angle of the videolaryngoscope blade to site with the larynx. An un-styleted ETT is unable to do this as its inherent radius of curvature of nearly 14 cm [9] cannot align with the acute radius of curvature of the D-blade of the C-Mac™ or other videolaryngoscopes, which is 7 to 8 cm (Figure 1b). This may result in a longer intubation time [10]. It is recommended that a pre-shaped, styleted tube be used during endotracheal intubation with a videolaryngoscope to overcome this problem [11]. The Glide-Scope™ and TruView™ PCD have their own pre-shaped dedicated rigid stylets, which do not permit reshaping. Unfortunately, the pre-shaping of the ETT with a stylet may not always suit an individual patient’s need [12]. In such a patient, if the stylet permits, such as the malleable Portex™ intubation stylet (PIS) (Smiths Medical ASD, Inc. Norwell, MA, USA], the ETT-stylet assembly has to be taken out and re-shaped before making another attempt at tracheal intubation. This may delay endotracheal intubation with a potential increased hemodynamic response and soft-tissue trauma [13,14].

**Figure 2:**
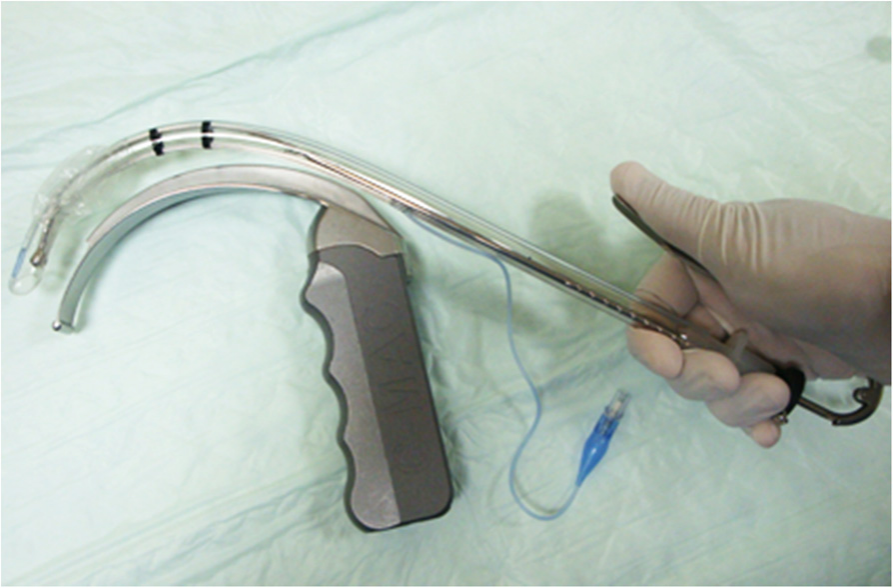
**Advantage of the Truflex™ articulating stylet.** The shape of the endotracheal tube has been changed to conform to that of the D-Blade using the Truflex™ articulating stylet.

The present trial (ISRCTN57679531) aims at utilizing the Truflex™ articulating stylet (TAS) (Truphatek) as a dynamic aid to tailor the ETT shape in its very first attempt as per the videolaryngoscope blade design (Figure 2) and the patient’s oropharyngeal anatomy. We hypothesize that a pre-loaded ETT over the TAS will not only shorten the D-blade C-Mac™ videolaryngoscope-aided intubation time, but will also attenuate the hemodynamic response and reduce the possibility of soft-tissue trauma, both in patients with normal anatomy and in those whose anatomy makes access more difficult, compared to non-dynamic PIS-aided tracheal intubation.

## Methods

**Figure 3:**
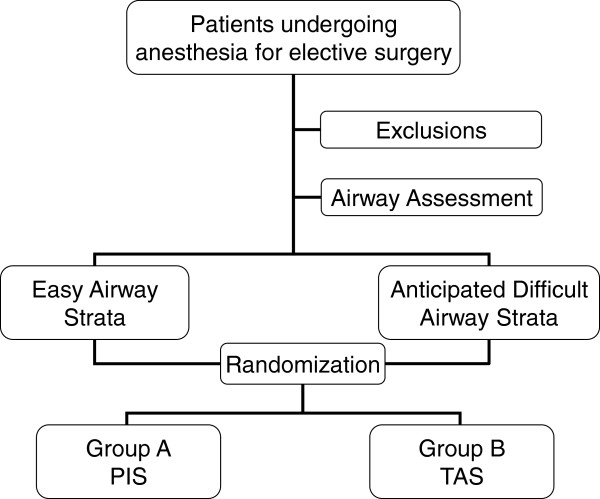
**Scheme of stratified randomization.** After appropriate exclusions all eligible patients will undergo airway assessment with the ADA score and stratified into easy and difficult airway strata, and will then be randomized into the two treatment groups. ADA, anticipated difficult airway; PIS, Portex™ intubation stylet; TAS, Truflex™ articulating stylet.

The study protocol has been approved by the Ethical Issues Committee, Khoula hospital. The current study is an interventional, single-blinded, parallel assignment, randomized controlled trial. The aim of the study is to compare the conventional rigid Portex™ stylet with the Truflex™ articulating stylet as an intubation guide during videolaryngoscopy. Stratified block randomization with variable block sizes will be done (Figure 3). The freeware Random Allocation software of Mahmood Saghaei will be used for generating randomization sequences [15]. Stratification will be based on anticipated difficulty in intubation based on the anticipated difficulty airway (ADA) score (Table 1). Sealed opaque envelopes will be used for allocation concealment with sequential numbering within each stratum. For randomization the envelopes will be opened only after transporting the patient to the operating theater, thus, maintaining allocation concealment and only one envelope can be opened per patient.

**Table 1:** Anticipated difficult airway (ADA) score^*^

**Airway factors**	**Score**		
	**0**	**1**	**2**
**Mallampati classification**	Class I	Class II	Class III to IV
**Thyromental distance, cm**	>6.5	6.0 to 6.5	<6.0
**Head and neck movement, degrees**	>90	90	<90
**Body mass index, kg/m**^**2**^	<25	≥25	NA
**Buck teeth**	No	Mild	Severe
**Inter-incisor gap, cm**	>5.0	4.0 to 5.0	<4.0

^*^Easy airway strata: ADA score ≤6; difficult airway strata: ADA score >6; NA- Not applicable.

**Figure 4:**
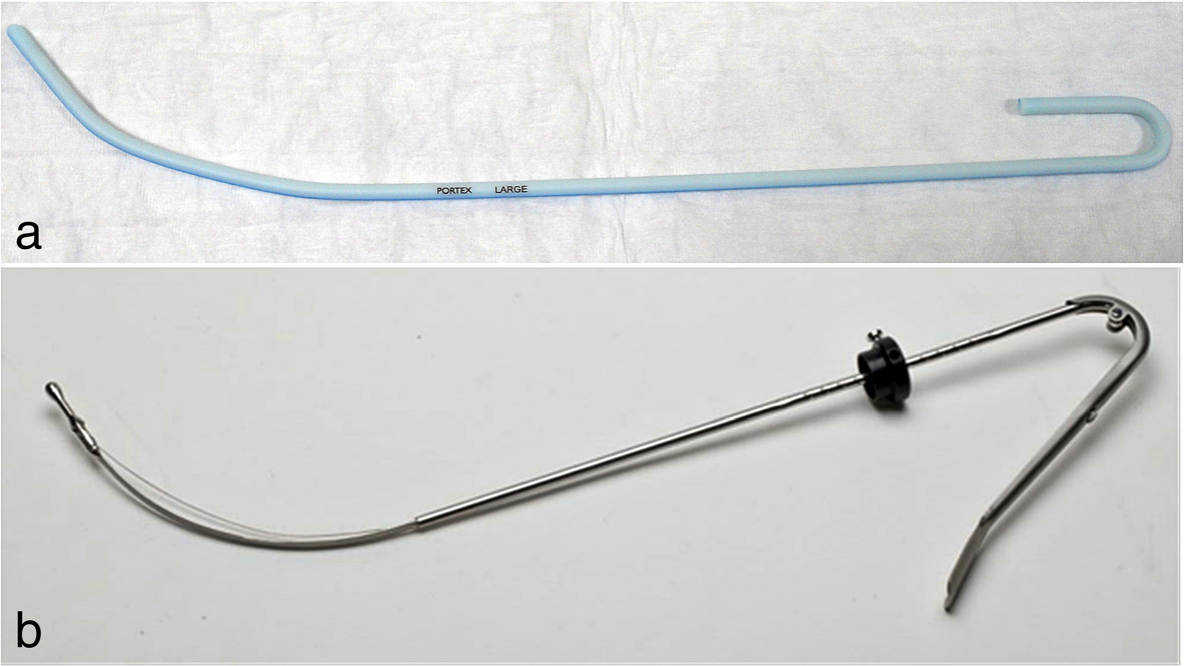
**Stylets used in the intervention arms. (a)** Conventional Portex™ intubation stylet used in group A patients. **(b)** Truflex™ articulating stylet with stopper used in group B patients.

Patients between 18 and 60 years of age, of either sex, graded I or II according to the criteria of the American Society of Anesthesiologists, undergoing elective surgical procedure under general anesthesia and tracheal intubation, and who give written informed consent, will be included. Patients with known airway pathology, previous ear, nose and throat (ENT) surgery of the oropharynx, known malignancies prior to randomization, immobilized cervical spine, known allergies to either study devices or its components, pregnancy, or known bleeding/coagulation disorder, will be excluded from the study. Patients will undergo a uniform induction technique with propofol 2.0 to 2.5 mg/kg and adequately relaxed with either cisatracurium 0.1 mg/kg or rocuronium bromide 0.6 mg/kg as evident by loss of all train of four responses using a peripheral nerve stimulator (Innervator Constant Current Peripheral Nerve Stimulator, Fisher & Paykel Health Care System, New Zealand). With the induction of anesthesia, patients shall also be administered 1.5 μg/kg of fentanyl. For patients in group A, a well-lubricated PIS (Figure 4a) will be used to shape the ETT according to the curvature of the videolaryngoscope D-blade. This pre-shaped ETT will be guided into the trachea after obtaining an adequate view of the glottis of an anesthetized and fully relaxed patient using the C-Mac™ D-blade videolaryngoscope. For patients in group B, a well-lubricated TAS (Figure 4b) will be used in place of the rigid stylet to change the curvature of the ETT as per need to negotiate into the glottis, using the same videolaryngoscope. In both groups, videolaryngoscopy and tracheal intubation will be done by an experienced anesthesiologist. An experienced anesthesiologist will be defined for the purpose of this study as an operator performing at least 300 videolaryngoscopies per year at a center with a case load of at least 500 videolaryngoscopies per year.

Primary efficacy endpoints will be successful or failed tracheal intubation in the first attempt, total intubation time and the intubation difficulty score. An attempt will be counted if the laryngoscope or the ETT needs to be removed for re-oxygenation (drop in oxygen saturation by 5%) or for reshaping of the ETT. The total intubation time will be the sum of the glotticoscopy time (from introduction of the videolaryngoscope blade between the teeth to the best laryngeal view) and the ETT negotiation time (from receiving the styleted ETT in laryngoscopist’s hand to passage of the black line just beyond the vocal cord). Secondary efficacy endpoints will be overall user satisfaction graded from 1 to 10 (1 = very poor, 10 = excellent), Cormack and Lehane’s grading, glotticoscopy time and ETT negotiation time and total number of intubation attempts with a maximum of three attempts, after which an alternative technique will be used. Safety endpoints assessed will be dental and airway trauma (present or absent), hemodynamic disturbances (blood pressure and pulse rate would be recorded before intubation, 1 minute and 5 minutes post-intubation; ≥20% change from the baseline value will be considered clinically important), arrhythmias (absent or present; type if present), or cardiac arrest.

### Statistical considerations

For sample size assessment, a pilot study of 60 patients was conducted in which we observed that successful intubation could be achieved in the first attempt in 100% (30/30) of patients in group B (TAS) but was achieved in 90% (27/30) of group A (PIS). To detect a similar difference between the success of intubation (100 versus 90%) with a statistical power of 80% and 95% confidence interval and equal distribution of patients in both the groups, a total sample size of 154 was estimated for the present study using the open source browser-based calculator OpenEpi [16]. However, in light of the stratification used for randomization the final sample size of 158 shall be used with equal distribution. All statistical evaluations will be made at a significance level of 0.05 (two-sided). A two-sided, 95% confidence interval will be calculated for the mean difference between the treatment groups. The data will be analyzed on an intention-to-treat basis. No imputations will be used for any missing data. The primary efficacy variable of success/failure between the two groups will be analyzed using the chi square/Fisher’s exact test, whichever will be applicable. Binary logistic regression will also be used on the whole dataset to identify predictors of success/failure of intubation. For evaluation of intubation times and the intubation difficulty score, analysis of variance (ANOVA) will be used for comparison between both treatment groups. The intubation difficulty scale (IDS) will also be transformed into an ordinal variable (0, 0 to 5, >5) and multinomial logistic regression will be used to evaluate for possible predictors of a difficult intubation.

## Discussion

The last decade has seen the emergence of videolaryngoscopes that enable us to visualize the laryngeal structures on a high-resolution video screen. These new aids provide a superior view of the laryngeal structures in normal conditions, and in a plethora of pathological and challenging conditions. However, the negotiation of the ETT into the trachea may still be a challenge despite using a malleable rigid stylet to pre-shape the ETT. In such situations, if the pre-shaping has not been optimal, the ETT-rigid stylet assembly needs to be taken out of the oropharynx for re-adjustment of its shape before making another attempt at tracheal intubation.

To overcome the limitation of a malleable rigid stylet, Schroeder’s directional stylet has been used to guide the ETT towards the glottis while using the D-blade of the C-Mac™ videolaryngoscope [12]. However, Schroeder’s directional stylet has two limitations. First, its length is not sufficient, especially for the armored ETT to be fully mounted over it, and second, it helps to change the shape of the premounted ETT into a C shape rather than a curvature at its distal end where it is actually needed. In contrast, the TAS has sufficient length and its shape can be molded at its distal end to the need of laryngoscopy and tracheal intubation.

TAS is a newly introduced device that has an easily controllable flexible tip using a lever that allows an upward movement of 30 to 60° at its distal 3 cm. The ETT is premounted over a well-lubricated TAS and a stopper at the proximal end of TAS helps to hold the ETT in position (Figure 4b). The TAS permits dynamic shaping of the curvature of the distal end of the premounted ETT by an angle as per need during videolaryngoscopy.

In conclusion, in this clinical trial, we are evaluating the efficacy of TAS in aiding tracheal intubation using the D-Blade of the C-Mac™ videolaryngoscope, using a randomized and single-blinded design. The success of the trial will significantly improve the application of this relatively new method of tracheal intubation.

## Trial status

The first participants were included on 3 March 2013. There were 11 patients recruited at the time this paper was submitted.

## Abbreviations

ADA score: Anticipated difficult airway score; ANOVA: Analysis of variance; ENT: Ear nose and throat; ETT: Endotracheal tube; IDS: Intubation difficulty scale; PCD: Picture capture device; PIS: Portex™ intubation stylet; TAS: Truflex™ articulating stylet.

## Competing interests

The authors claim no conflicts of interest.

## Authors' contributions

AA-Q was involved with the study plan, data collection, and approval of the manuscript. WA-A was involved with planning of the study, data collection, and approval of the manuscript. MA helped design the study, plan the statistical analysis, and helped write the manuscript. RMK helped design the study, performed videolaryngoscopies, and helped write the manuscript. NK helped design the study, performed videolaryngoscopies, and helped write the manuscript. All authors read and approved the final manuscript.
